# Coronavirus Disease 2019 Outbreak in a Psychiatric Closed Ward: What We Have to Learn

**DOI:** 10.3389/fpsyt.2020.579235

**Published:** 2021-01-22

**Authors:** June Young Chun, Jin Yong Jun, Jiah Choi, Minho Jo, Kyongmin Kwak, Yunjin Jeong, Junsoo Lee, Junkyu Park, Kang Hee Lee, Yoonyoung Nam, Young Ju Choi, Young Moon Lee, Nam Joong Kim

**Affiliations:** ^1^Department of Internal Medicine, National Cancer Center, Goyang, South Korea; ^2^Department of Psychiatry, National Center for Mental Health, Seoul, South Korea; ^3^Department of Internal Medicine, Keimyung University, Daegu, South Korea; ^4^Department of Internal Medicine, Sangju Redcross Hospital, Sangju, South Korea; ^5^Public Health Center, Ministry of Health and Welfare, Seoul, South Korea; ^6^Department of Planning and Public Relations, National Center for Mental Health, Seoul, South Korea; ^7^Department of Internal Medicine, Seoul National University College of Medicine, Seoul, South Korea

**Keywords:** COVID-19, SARS-CoV-2, infectious disease transmission, psychiatric hospital, schizophrenia

## Introduction

As of December 1, 2020, 34,652 coronavirus disease 2019 (COVID-19) cases have been identified in South Korea (1). Several clusters, especially in Daegu City and North Gyeongsang Province, caused a surge in COVID-19 cases. In February of 2020, an outbreak occurred with an extremely high attack rate in a psychiatric closed ward in North Gyeongsang Province. A total of 102 (98.1%) of 104 patients who had been staying in the closed ward were diagnosed with COVID-19. Here, we briefly report this outbreak, explore the contributing factors of the high attack rate, and further suggest infection prevention measures in psychiatric closed wards.

## Outbreak Investigation

When a COVID-19 outbreak in the psychiatric hospital was noticed, psychiatrists and physicians of the Ministry of Health and Welfare were dispatched to triage patients. They classified patients according to severity and transferred them to other nationally designated hospitals. The hospital where the outbreak occurred was closed down. The National Center for Mental Health (NCMH) is a 240-bed teaching hospital in Seoul, an institution affiliated with the Ministry of Health and Welfare of South Korea. Sixty-four patients from the psychiatric ward were transferred to the NCMH from February 26 to April 13, 2020, and were included in this study. Baseline characteristics, such as age, sex, and underlying diseases, were gathered by the dispatched clinicians in the psychiatric hospital with the COVID-19 outbreak. Laboratory and radiologic findings were collected in the NCMH. The study was approved by the Institutional Ethics Review Board of the NCMH (No. 116271-2020-25).

## Epidemiologic Characteristics

On February 15, 2020, patients in the closed ward presented with fever and upper respiratory tract symptoms simultaneously. An attending physician suspected an influenza outbreak and performed influenza rapid antigen tests. However, none of them tested positive, and one patient died of pneumonia of unknown etiology on February 19. The cause of death was COVID-19, which raised the concern of a COVID-19 outbreak in the closed ward. Diagnostic tests for COVID-19 were performed for all inpatients on February 21. Of the 103 remaining patients other than the first deceased patient, 101 were positive for severe acute respiratory syndrome coronavirus 2 (SARS-CoV-2) nucleic acid tests, resulting in the secondary attack rate of 98.1%. Among the 102 confirmed patients (including one deceased patient), seven died of COVID-19 by April 20, 2020, resulting in the case fatality ratio of 6.9%. The index case could not be identified because patients came in contact with each other, and an ideal history-taking was difficult for their underlying diseases. A closed unit of the psychiatric ward was placed on the top floor of the four-story hospital. A total of 104 patients with psychiatric disorders stayed in 15 rooms. All windows were sealed owing to the concern of self-injury, and the patients stayed in the multi-sharing rooms for a long time.

## Clinical Characteristics

The mean (±standard deviation) age of the 64 patients was 56.7 ± 9.3 years, and the male-to-female ratio was 1.7:1. The most common mental illness was schizophrenia, which accounted for approximately 79.7% (51/64) of patients, followed by intellectual disability (7.8%, 5/64) (Table 1). Hypertension and diabetes were the most common underlying diseases, accounting for 51.6% (33/64) and 29.7% (19/64) of the total cases, respectively. The median hospitalization period of these patients at a psychiatric closed ward was 4.5 years (interquartile range, 2–6 years). Fever, cough, and sputum were noted in patients, although the exact proportions had not been documented. Thirty patients (46.9%) had radiologic signs of pneumonia, of which seven patients had received oxygen therapy. Thirty-five patients (54.7%) received antiviral agents, including lopinavir/ritonavir and hydroxychloroquine.

**Table 1:** Baseline and clinical characteristics of 64 patients with COVID-19.

**Characteristics**	**Value**
Age^a^	56.69 ± 9.25
Sex (male)	40 (62.5%)
Body mass index (kg/m^2^)^a^	24.51 ± 5.00
Mental illness^*^	
Schizophrenia	51/64 (79.7%)
Bipolar disorder	3/64 (4.7%)
Alcohol use disorder	3/64 (4.7%)
Organic mental disorder	3/64 (4.7%)
Mental retardation	5/64 (7.8%)
Dementia	3/64 (4.7%)
Medical illness^*^	
Hypertension	33/64 (51.6%)
Diabetes	19/64 (29.7%)
Heart disease	2/64 (3.1%)
Chronic renal disease	1/64 (1.6%)
Chronic liver disease	5/64 (7.8%)
Chronic lung disease	8/64 (12.5%)
Cancer	0/64 (0.0%)
**Blood cell counts^b^**	
White blood cells (/μl)	4,500 (3,738–5,243)
Lymphocytes (%)	33.2 (26–41.2)
Hemoglobin (g/dl)	12.6 (11.6–13.6)
Platelets (/μl)	198,000 (153,250–251,500)
**Blood biochemistry^b^**	
Blood urea nitrogen (mg/dl)	10.4 (7.63–13.5)
Serum creatinine (mg/dl)	0.9 (0.7–1.1)
Total protein (g/dl)	6.8 (6.5–7.2)
Albumin (g/dl)	4 (3.7–4.2)
Cholesterol (mg/dl)	138 (121–172)
Radiologic signs of pneumonia (%)	30/64 (46.9%)
**Treatment**	
Antiviral treatment^§^	35/64 (54.7%)
Oxygen therapy	7/64 (10.9%)
ECMO	1/64 (1.6%)

*COVID-19, coronavirus disease 2019; ECMO, extracorporeal membrane oxygenation*.

a *Data are presented as mean ± SD*.

b *Data are presented as median (interquartile range); otherwise, n (%), n/N (%)*.

* *Duplication allowed*.

§ *Includes lopinavir/ritonavir and hydroxychloroquine*.

## Discussion

Nosocomial transmissions of SARS-CoV-2 have been reported among patients and health-care workers in hospitals, nursing centers, and daycare centers (2–4). However, this outbreak was extremely unusual in several aspects. First, the secondary attack rate was 98.1% (102/104), which was much higher than any others reported. The attack rate of SARS-CoV-2 nosocomial transmission ranges from 0 to 64% (5). Second, the case fatality ratio was relatively high considering the age distribution of the patients. The case fatality ratio was highly variable between countries and ranged from 0.0 to 17.7%, with a global average of 6.8% (6). The case fatality ratio in this study population was 6.9% (7/102), which was higher than that in South Korean patients with COVID-19 (2.4% as of May 16, 2020) (7). The case fatality ratio among patients in their 50s in a psychiatric ward and nationwide was 9.3% (4/43) and 0.8% (15/1,951), respectively, which showed a significant difference (Fisher's exact test, *p* < 0.05) (7).

The secondary attack rate is defined as the probability that an infection develops among susceptible individuals within a specific group. We speculate several reasons for such a high attack rate and case fatality ratio in this outbreak. First, unfavorable environment in terms of infection prevention may have contributed to this high attack rate. There was no concept of a designated bed for a person, and patients came in contact with one another and shared the space together. The average bed space per individual was <4.3 m^2^, which did not meet the standard space requirement of 6.3 m^2^ in South Korea, and was far smaller than the required 9.29 m^2^ in the United States or 13.32 m^2^ in the United Kingdom (8, 9). All doors and windows were closed owing to concerns about escape or self-injury. Alcohol-based hand sanitizers were not available in the closed ward because of the possibility that patients may drink the alcohol by accident. Second, hospitalization periods were extremely long for most patients. We assume that a long stay in a closed ward could cause generalized weakness and render them more vulnerable to COVID-19. Lastly, patients with mental illness could not explain their symptoms effectively. This could contribute to the delay in the diagnosis of COVID-19. Thirty of the 64 patients had pneumonia, and the proportion of patients who initially presented with radiologic pneumonia was relatively high, which suggests a diagnostic delay.

There was another report of a COVID-19 outbreak in a psychiatric hospital in Wuhan city; at least 50 inpatients with mental health disorders and 30 physicians were diagnosed in a 950-bed hospital (10, 11). In the United States, more than 1,450 COVID-19 cases at state mental health facilities were identified in 23 states and Washington, DC (12). The similar factors of COVID-19 outbreaks in psychiatric hospitals from several countries were (i) confined conditions in psychiatric wards, (ii) lack of keen medical intervention, and (iii) vulnerable patient factors such as accompanying chronic medical illness and cognitive impairment that made them unaware the risk or to explain their symptoms effectively. In this regard, we suppose that an emerging infectious disease could become a much bigger threat to individuals with severe mental illness, possibly exacerbating already existing health inequalities (13, 14). As described above, median hospitalization period in this study was 4.5 years, which must have been longer than those of other outbreaks. We suggest that long stay in a hospital made them more susceptible to infectious diseases.

We herein suggest several preferable measures for infection prevention in psychiatric closed wards from what we have learned. First, considering that closed wards are inevitably difficult to ventilate, the structural systems of heating, ventilating, and air-conditioning (HVAC system) must be reinforced, especially in psychiatric facilities. The use of highly efficient filtration in HVAC systems could reduce the transmission of infectious particles in closed units. Second, controlling visitor access would be necessary, particularly during an outbreak. It is unlikely that any inpatient would be the index case, since patients have limited social contact. Rather, health-care workers, voluntary workers, or any visitors may be the likely source of the outbreak. Routine temperature checks, informing visitors not to enter closed wards if ill, wearing a face mask, and sanitizing hands for all entries would prevent most transmission. Third, a prompt response to febrile patients in psychiatric wards would be important. When numerous febrile patients were noticed and the influenza antigen results were negative for all, it was reasonable to suspect COVID-19, considering the increasing number of cases in neighboring areas. These patients may not be able to draw a physician's attention because they cannot express their symptoms in detail. Preemptive isolation, testing, and treatment could result in favorable outcomes. Lastly, provision of proper mental health services would be as important as medical health for patients with severe mental illness (15). Focusing on the unprecedented outbreak might be a pressure on health-care systems and probably hinder the proper mental health care, which in turn worsen the outbreak. Coordinated medical and mental health interventions would be in necessity (15).

We must consider that the influx of COVID-19 into a closed ward could cause an extremely high attack rate and should be more vigilant against COVID-19 in psychiatric wards.

## Ethics Statement

The study was approved by the Institutional Ethics Review Board of NCMH (No. 116271-2020-25). Written informed consent for participation was not required for this study in accordance with the national legislation and the institutional requirements.

## Author Contributions

YL and NK designed the study. JC and JJ drafted the manuscript. All authors contributed to the article and approved the submitted version.

## Conflict of Interest

The authors declare that the research was conducted in the absence of any commercial or financial relationships that could be construed as a potential conflict of interest.
